
JOURNAL INFORMATION
==============================
NLM Title Abbreviation: Buffalo Med J
NLM Journal ID: 0027534

Buffalo Medical Journal

ISSN: 1040-3817
Publisher: Biomedical Journal Digitization Project

ARTICLE INFORMATION
==============================
PMCID: PMC8726957
PMID: 36885319
Article version: 1
Subjects: Items

Items

Print publication date: 1908 Sep
Volume: 64
Issue: 2
First page: 120
Last page: 120
License: This work is free of known copyright restrictions. For more information, please see PMC Back Issue Digitization.
License URL: https://creativecommons.org/publicdomain/mark/1.0/

==============================

### ITEMS.

BATTLE & COMPANY, Saint Louis, have issued No.6 of a series of eighteen illustrations in colors, showing as many dislocations of important joints. The present number graphically portrays a subglenoid dislocation of the humerus. It will be sent to physicians on application to the publishers.

THE Antikamnia Chemical Company, now located at 1624 Pine street, St. Louis, will begin September 1, 1908, the construction at the northeast corner of Pine and Fourteenth streets of a five-story and basement building which will be used for manufacturing and commercial purposes. It will have a ground area of 81x109 feet, the latter frontage being on Fourteenth street, and will be constructed of brick and concrete. It will be modern in every detail, being provided with sprinkler system, fast elevators, etc., and will cost in the neighborhood of $75,000. The site has been owned by Mr. Frank A. Ruf, president of the company and a director in the Mercantile Trust Company for the past seven years.

The Antikamnia Company will occupy all of the new structure, the upper floors being used for a can and box factory. Its present quarters at 1624 Pine street are inadequate and the property on either side of it is so tied up that it can not be secured to extend the building. On this account the company expects to give up the building at the expiration of its present lease, which has a year or more to run, and to make the structure at Fourteenth and Pine its headquarters.

**MEDICAL SUPERINTENDENT, CRAIG COLONY FOR EPILEPTICS, SONYEA, N. Y.**

A competitive civil service examination for this position, open to residents of New York State, will be held September 26, 1908. For full particulars and application forms, address **Chief Examiner,** State Civil Service Commission, Albany, N. Y.

FOR RENT—Partly furnished if desired, and with immediate possession: Suite of Offices at 217 Franklin Street for **Physician,** containing five large rooms, and bath with hot water; also having skylighted operating room, with separate rear entrance and every modern convenience.

This suite is on ground floor, the location unsurpassed, surroundings in every way unobjectionable, and the opportunity considered rare and desirable and one that is seldom offered.

Apply—F. W. HUMBLE,
409 Franklin Street.
